# Transformer Dil-DenseUnet: An Advanced Architecture for Stroke Segmentation

**DOI:** 10.3390/jimaging10120304

**Published:** 2024-11-25

**Authors:** Nesrine Jazzar, Besma Mabrouk, Ali Douik

**Affiliations:** 1 Research Laboratory: Networked Objects, Control and Communication Systems, NOCCS-ENISo, National Engineering School of Sousse, University of Sousse, Soussse 4023, Tunisia; ali.douik@eniso.u-sousse.tn; 2 National Engineering School of Sfax, University of Sfax, Sfax 3038, Tunisia; 3 Research Laboratory: Advanced Technologies for Medicine and Signals ATMS, Department of Electrical and Computer Engineering, National Engineers School, University of Sfax, Sfax 3038, Tunisia; mabroukbesma@ymail.com

**Keywords:** stroke image segmentation, MRI, DenseUNet, dilated convolution, Transformer block

## Abstract

We propose a novel architecture, Transformer Dil-DenseUNet, designed to address the challenges of accurately segmenting stroke lesions in MRI images. Precise segmentation is essential for diagnosing and treating stroke patients, as it provides critical spatial insights into the affected brain regions and the extent of damage. Traditional manual segmentation is labor-intensive and error-prone, highlighting the need for automated solutions. Our Transformer Dil-DenseUNet combines DenseNet, dilated convolutions, and Transformer blocks, each contributing unique strengths to enhance segmentation accuracy. The DenseNet component captures fine-grained details and global features by leveraging dense connections, improving both precision and feature reuse. The dilated convolutional blocks, placed before each DenseNet module, expand the receptive field, capturing broader contextual information essential for accurate segmentation. Additionally, the Transformer blocks within our architecture address CNN limitations in capturing long-range dependencies by modeling complex spatial relationships through multi-head self-attention mechanisms. We assess our model’s performance on the Ischemic Stroke Lesion Segmentation Challenge 2015 (SISS 2015) and ISLES 2022 datasets. In the testing phase, the model achieves a Dice coefficient of 0.80 ± 0.30 on SISS 2015 and 0.81 ± 0.33 on ISLES 2022, surpassing the current state-of-the-art results on these datasets.

## 1. Introduction

Stroke remains one of the most debilitating neurological disorders globally, affecting approximately 12.2 million individuals each year, with over 7.8 million deaths and 5 million cases of permanent disability [1]. Stroke occurs when the blood flow to specific areas of the brain is interrupted, leading to rapid cell death due to the lack of oxygen and nutrients. Timely diagnosis and intervention are crucial, as early treatment can significantly improve patient outcomes. Advanced imaging techniques, such as Positron Emission Tomography (PET), Computed Tomography (CT), CT perfusion (CTP), and Magnetic Resonance Imaging (MRI), provide valuable information about stroke lesions, including their shape, size, location, and metabolism. Among these, MRI is particularly favored due to its sensitivity to early tissue changes, making it essential for accurate diagnosis and effective treatment planning [2]. As a major health burden, stroke also imposes an economic cost of approximately USD 100 billion annually on the global economy.

Automated stroke lesion segmentation from brain imaging has become a key component of clinical workflows, aiding in lesion assessment, diagnosis, and treatment decision-making. Over the past decade, significant progress has been made in this area, particularly with the advent of deep learning models such as convolutional neural networks (CNNs). These models, including U-Net [3], have been successful in identifying complex patterns in medical images. However, CNNs have limitations, especially in capturing long-range dependencies crucial for accurately delineating stroke lesions, which often vary significantly in size, shape, and location [4]. To address this, recent work has explored hybrid models that combine the strengths of different architectures. Vision Transformers (ViTs) [5,6,7] have shown promise in capturing long-range spatial dependencies through multi-head self-attention mechanisms, originally developed for natural language processing [8], and have been increasingly applied to medical image segmentation tasks.

Despite these advancements, challenges remain in stroke lesion segmentation, particularly in cases with low contrast, diffuse boundaries, and irregular lesion shapes. In this work, we propose Transformer Dil-DenseUNet, a novel architecture that combines the strengths of DenseNet’s dense connectivity, dilated convolutions, and transformers to address these challenges and enhance stroke lesion segmentation accuracy. DenseNet’s dense connections promote effective feature reuse and capture both global and fine-grained details. Dilated Dense Blocks (DDBs) extend the receptive field without adding significant computational cost, allowing the model to capture critical contextual information necessary for accurate lesion delineation. The integration of transformers in the network enables the capture of long-range dependencies, improving segmentation precision even in low-contrast regions. Our results demonstrate that Transformer Dil-DenseUNet outperforms current state-of-the-art methods, particularly in capturing complex lesion details, as evidenced by higher Dice scores and improved segmentation accuracy on the MICCAI ISLES SISS 2015 [9] and ISLES 2022 datasets [10].

The paper’s contribution can be summarized as follows.

- Development of Transformer Dil-DenseUNet: A novel hybrid architecture integrating DenseNet, dilated convolutions, and transformers to improve stroke lesion segmentation accuracy.
- Enhanced Contextual Understanding: The use of Dilated Dense Blocks (DDBs) expands the receptive field efficiently, improving the model’s ability to handle challenging low-contrast regions and irregular lesion boundaries.
- Superior Performance Validation: Our model achieves state-of-the-art results on both the MICCAI ISLES SISS 2015 and ISLES 2022 datasets, surpassing existing models in segmentation accuracy and Dice scores. Ablation studies confirm the effectiveness of each architectural component.

The remainder of this paper is organized as follows: Section 2 reviews related work, focusing on recent advances in hybrid models for medical image segmentation. Section 3 details the architecture and training methodology. Section 4 outlines the datasets and preprocessing techniques. Section 5 presents the evaluation metrics and experimental results. Finally, Section 6 concludes the study.

## 2. Related Works

Accurate stroke lesion segmentation in MRI scans remains a significant challenge in clinical practice. To address this, researchers have developed various deep learning models, particularly convolutional neural networks (CNNs), such as U-Net, that aim to enhance segmentation efficiency while managing computational complexity. These models often incorporate advanced modules, such as residual [11], dense [12], dilated [13], and attention [14], to improve accuracy, particularly for detecting small or subtle lesions.

Recent advancements in stroke lesion segmentation can be broadly categorized into convolutional-based approaches and transformer-enhanced models. Convolutional methods continue to be popular for capturing local spatial features, while transformer-based models, which excel at modeling long-range dependencies and global context, offer a complementary approach for addressing the complex patterns of stroke lesions in MRI. Both categories strive to improve segmentation performance, yet each faces challenges, such as high computational costs, limited dataset availability, and difficulties in detecting small or fragmented lesions.

### 2.1. Convolutional-Based Approaches

Convolutional architectures remain foundational for segmentation tasks due to their ability to capture spatial hierarchies effectively. Liu et al. [15] introduced the Deep Residual Attention Network (DRANet), a U-shaped model with dual-branch attention designed to enhance ischemic stroke lesion segmentation by improving lesion feature distinction. This attention module includes a main branch and a dilated soft mask branch, which collaboratively highlight key areas, enabling the network to better capture relevant lesion features. Evaluated on the SISS 2015 dataset, the model achieved a Dice coefficient of 0.76, though improvements are still needed for accurately segmenting smaller, scattered lesions. Similarly, Kumar et al. [16] introduced the Classifier–Segmenter Network (CSNet) to enhance segmentation accuracy. The CSNet model features a segmentation module made up of convolutional layers combined with batch normalization and ReLU activation, refined and validated through five-fold cross-validation. This model builds on a self-similar U-Net framework tailored to segmentation tasks. Tested with the ISLES SISS 2015 dataset, CSNet reached Dice scores of 0.63 and 0.83 on the SPES dataset and a Dice score of 0.28 with the ISLES 2017 dataset. Despite its effectiveness, the approach has notable limitations, such as its reliance on high-quality labeled datasets and the substantial computational power needed, which can restrict accessibility to high-performance hardware. Clèrigues et al. [17] proposed a deep learning approach using a 3D U-Net with an asymmetric encoder–decoder structure for segmenting acute and sub-acute stroke lesions from multimodal MRI data. This method addresses class imbalance through small patches and dynamically weighted loss functions, enhancing lesion detection. Additionally, brain symmetry augmentation leverages anatomical symmetry to improve feature extraction and lesion localization. Validated on the ISLES 2015 dataset, the model demonstrated strong performance, achieving a Dice score of 0.59 in the SISS challenge. Nevertheless, this approach requires the careful tuning of patches and symmetry strategies to maintain robustness across various lesion types.

Convolutional-based approaches generally excel in capturing local features but struggle with global context, especially in complex lesion patterns. Furthermore, many models depend heavily on high-quality labeled datasets and substantial computational resources, limiting their accessibility and applicability in resource-constrained settings.

### 2.2. Transformer-Enhanced Methods

Recent work has integrated self-attention mechanisms to capture long-range dependencies and global context in medical images. Alshehri et al. [18] proposed an approach that uses few-shot learning in combination with a CNN enhanced by self-attention to facilitate automatic segmentation of ischemic stroke lesions in MRI images. This method tackles the common issue of limited annotated medical datasets by implementing prototypical networks (PNet), which streamline few-shot learning and reduce the dependency on large labeled datasets. By integrating the FLAIR and DWI modalities at an early stage, the approach significantly improves the representation of features, resulting in higher segmentation accuracy. In evaluations on the SSIS 2015 dataset, the method achieved a Dice coefficient of 0.68. However, some challenges remain, particularly in the system’s ability to detect very small lesions. Wang et al. [19] developed an architecture called Multi-Encoder Transformer (METrans), designed to extract features at different stages of the encoder by integrating multiple encoding modules. This approach combines these multi-scale features to improve performance. To further enhance feature extraction, Convolutional Block Attention Modules (CBAMs) are applied after each convolutional block, utilizing both spatial and channel attention mechanisms. Additionally, a transformer module is added at the bottleneck to effectively capture global features. The method achieved a Dice coefficient of 0.79 when validated on the ISLES 2015 dataset. Wu et al. [20] present W-Net, an innovative two-stage network specifically designed for ischemic stroke lesion segmentation using multimodal MRI data. This model combines a CNN-transformer architecture and includes a boundary deformation module (BDM) that enhances boundary estimation for accurate lesion segmentation. W-Net achieves notable performance, with a Dice coefficient of 0.61 on both the ATLAS v1.2 and ISLES 2022 datasets. Dogru et al. [21] proposed a modified recurrent U-Net model for automated segmentation of ischemic stroke lesions from multi-spectral MRI scans. The model utilizes a five-layer architecture with recurrent convolutions incorporated after concatenation using dilated convolution layers. This adjustment helps detect stroke information in small, fragmented regions while maintaining computational efficiency. The model was trained using a leave-one-out cross-validation approach on MRI slices from the ISLES 2015 and ISLES 2022 datasets, achieving a Dice coefficient of 0.748. However, challenges remain, especially in detecting fragmented, small lesions, as evidenced by lower performance in these cases. Zafari-Ghadim et al. [22] reviewed various Transformer-based architectures for stroke lesion segmentation, emphasizing their ability to capture long-range dependencies and complex spatial patterns. Several architectures integrate CNNs and Transformers to benefit from both local feature extraction and global context modeling. Models like UCATR and UTransNet leverage hybrid CNN–Transformer frameworks to segment ischemic strokes in MRI and CT images, achieving Dice scores around 0.74–0.79. However, pure Transformer models face limitations, including high computational demands and challenges with 3D medical images. Despite these advancements, the accurate detection of smaller lesions and the handling of image heterogeneity remain challenging.

While Transformer-based methods are effective at capturing long-range dependencies and global context, they often face challenges related to high computational costs and difficulties in handling 3D medical images. Additionally, small lesion detection and managing heterogeneous data remain significant issues.

These studies collectively underscore the growing interest in and exploration of DensUNet and Transformer architectures for medical image segmentation tasks, showcasing their potential to outperform CNN methodologies and advance the state-of-the-art in stroke diagnosis and treatment.

## 3. Methods

### 3.1. Overview of Our Model

**Figure 1:**
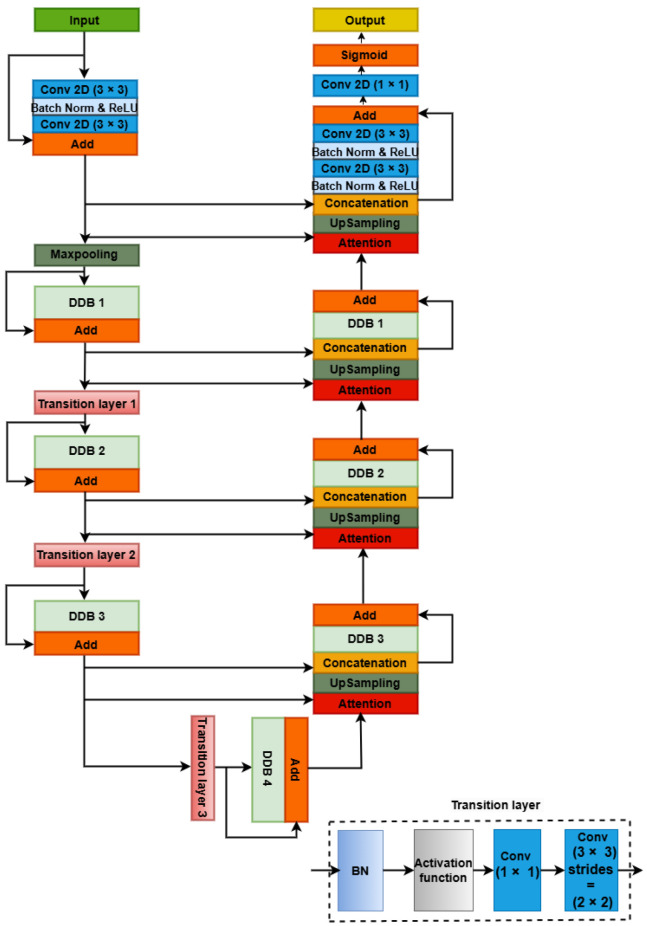
Proposed Transformer Dil−DenseUNet architecture for stroke image segmentation.

The architectural design in Figure 1 presents Transformer Dil-DenseUnet, an encoder-decoder model specifically crafted for stroke image segmentation. This architecture combines DenseUNet, dilated convolutions, and Transformers to capture both fine local details and broader contextual information, which is essential for accurately identifying stroke lesions. The encoder pathway reduces image dimensionality to extract high-level, stroke-specific features, while the decoder pathway progressively upscales and refines these features, producing a precise segmentation mask tailored to stroke pathology. Our proposed model contains one stem block, followed by three DDB blocks in the encoder part, a DDB in the center, and three DDB blocks in the decoder part. Two transition layers are incorporated into the encoder and one transition layer in the bottleneck to enhance feature processing across stages.

**Figure 2:**
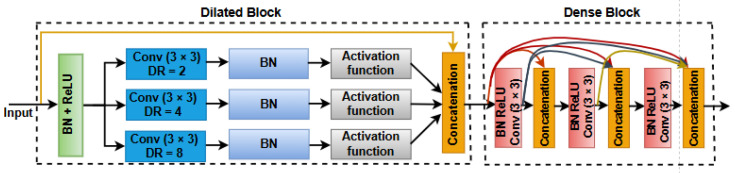
Diagrammatic representation of the DDB block.

Each DDB consists of a dilated block on the left and a Dense Block on the right, as depicted in Figure 2. Positioned on the left side, the dilated block encompasses a broad receptive field, empowering it to grasp extensive contextual information. In the dilated block, there are three convolutional layers, employing a kernel dimension of 3 × 3 and a dilation rate that doubles in each layer (DR = 2, 4, 8). Conversely, the dense block, situated on the right, generates a feature map that encapsulates highly accurate target details as it traverses multiple layers. To leverage the advantages of a broader receptive field while maintaining the intricate details of the target information, we strategically position the Dense Block after the dilated block. This arrangement facilitates the seamless inheritance of the contextual influence garnered by the dilated block. The dense block comprises a series of concatenation layers that combine the outputs from preceding layers with the current layer’s output. This dense connectivity is thought to exploit efficient information propagation during both forward and backward passes. The dense block itself comprises batch normalization (BN), rectified linear unit (ReLU), and 3 × 3 convolution (Conv) operations. This compositional structure ensures effective feature extraction and integration within the Dense Block.

The transition layer is an essential component of our model, designed to optimize feature extraction and improve overall performance. Located exclusively in the encoder pathway and bottleneck sections, the transition layer performs three core functions:

1. Dimensionality Reduction: By compressing feature maps, the transition layer retains critical information while reducing computational complexity, which is essential as the feature depth increases. This dimensionality reduction allows for efficient processing without compromising essential details.
2. Enhanced Feature Propagation: After each Dense Block in the encoder, the transition layer consolidates features via batch normalization, ReLU activation, and 1 × 1 convolution. These operations promote smooth feature flow, stabilize the network during training, and emphasize critical patterns, enhancing the model’s feature propagation capability.
3. Improved Spatial Understanding: Incorporating a strided 3 × 3 convolution, the transition layer halves the spatial dimensions, expanding the receptive field to capture larger contextual features in input images. This improves the model’s spatial understanding, which is especially crucial for capturing details in medical images and enhances segmentation performance.

The decoder pathway mirrors the encoder’s structure with three DDBs. However, instead of downsampling, the decoder utilizes upsampling operations after each DDB to enhance the resolution of the feature maps.

Crucially, skip connections probably exist between corresponding DDBs within both the encoder and the decoder pathways. These connections concatenate the upsampled feature maps with those from the corresponding encoder block. This strategy preserves spatial information lost during downsampling in the encoder, ultimately leading to more accurate segmentation results. The architecture incorporates an attention mechanism after each upsampling block. Attention mechanisms help the model prioritize the most pertinent features for segmentation within the upsampled feature maps, further improving segmentation accuracy.

**Figure 3:**
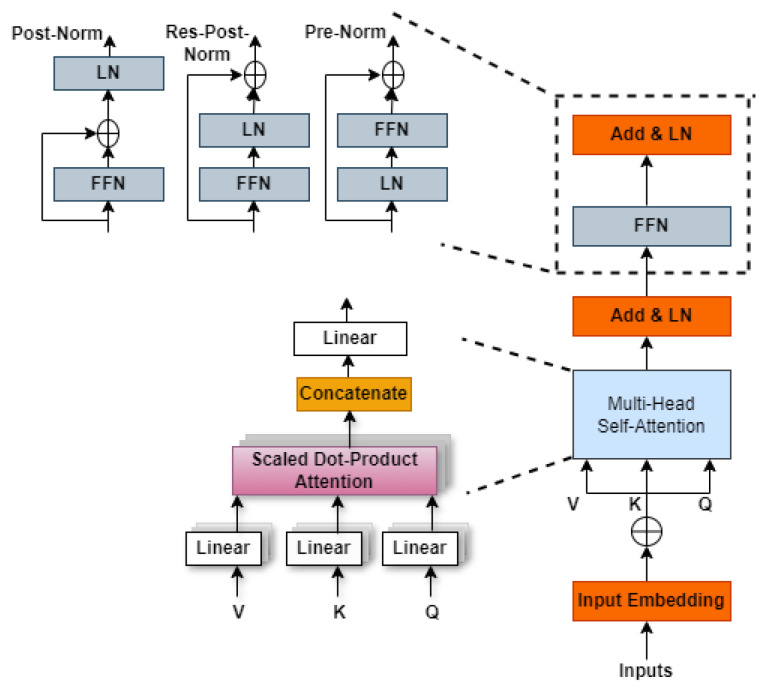
Illustration of a block diagram of a Transformer module.

The Transformer module, comprising two sub-layers, is depicted in Figure 3 (a figure from [23]): a feed-forward layer (FFN) and a multi-head self-attention layer. The multi-head self-attention layer enables the architecture to focus on various aspects of the input sequence. Following this, the feed-forward layer, a basic neural network, is employed to capture non-linear relationships within the sequence elements. Both sub-layers are supplemented by a residual connection and a normalization layer. This residual connection allows the model to grasp long-range dependencies within the sequence, while the layer normalization aids in stabilizing the training process.

Finally, the decoder’s output is likely passed through a sigmoid activation function to create a segmentation map. In the forthcoming subsections, we will provide concise explanations for each element within our proposed model.

#### 3.1.1. DenseUNet

An extension of DenseNet [23] fused with the U-Net architecture, DenseUNet presents a pioneering methodology for semantic segmentation tasks in medical imaging and beyond. It inherits DenseNet’s dense connectivity patterns, merging them with the expansive encoder and intricate decoder components of U-Net. This combination leverages the strengths of both architectures, effectively addressing the challenges inherent in stroke lesion segmentation, such as accurately delineating lesions and efficiently extracting relevant features from medical imaging datasets. By integrating these approaches, it improves the precision of lesion identification, particularly for complex and subtle stroke patterns.

One notable aspect of DenseUNet is its densely connected encoder–decoder structure. The encoder part efficiently extracts hierarchical features from input images through a series of densely connected Dense Blocks and downsampling operations, preserving fine-grained details while gradually reducing spatial dimensions. The decoder part then utilizes dense skip connections to recover spatial information lost during downsampling, facilitating precise localization of object boundaries in the segmentation maps.

Another notable aspect of DenseUNet is its use of residual connections within each Dense Block, similar to ResNet [24], to enhance the smoothness of gradient propagation and mitigate the vanishing gradient problem. By enabling direct paths for information flow between layers, residual connections promote stable training and help prevent the degradation of performance with increasing network depth.

Furthermore, we incorporate dilated convolutions within the Dense Block of DenseUnet to effectively capture multi-scale contextual information without a significant increase in computational complexity. This enables the model to incorporate a broader context while maintaining a large receptive field, enhancing its ability to grasp intricate local details alongside global context crucial for precise segmentation.

#### 3.1.2. Dilated Convolution

Dilated convolutions, also referred to as atrous convolutions, are a valuable technique in stroke lesion segmentation, particularly for capturing multi-scale contextual information. Unlike traditional convolutions, where filter elements are applied directly adjacent to each other, dilated convolutions introduce a dilation rate, which places gaps between the filter elements. This approach effectively increases the receptive field, allowing the model to capture broader contextual information from the input image without significantly increasing the number of parameters. This is especially important in stroke lesion segmentation, where it is essential to identify relationships between distant pixels to accurately delineate lesion boundaries. By incorporating dilated convolutions, the model can efficiently process larger regions of MRI scans, improving its ability to detect and segment ischemic lesions, even those that are sparse or fragmented. In our proposed architecture, dilated convolutions are strategically placed before the Dense Block, forming a Dilated Dense Block (DDB) that enhances the model’s ability to capture both local and global features for more precise lesion segmentation.

#### 3.1.3. Transformer Block

Attention mechanisms, widely recognized for their success in natural language processing (NLP) [25], have demonstrably improved performance in tasks like semantic segmentation, including pixel-wise prediction [26]. This technique empowers neural networks to selectively focus on crucial regions within the input data. This focus is particularly valuable for tasks like stroke segmentation in medical images, where identifying critical areas is essential. Transformers utilize a multi-head self-attention mechanism that excels at capturing long-range dependencies by integrating information from distant regions within an image. Long-range dependencies refer to the ability to relate and utilize information from far-apart areas within the data, which is particularly advantageous in stroke imaging. Here, lesions often present subtle boundaries and considerable variability in shape and size, making it essential to consider spatial context beyond the immediate surroundings. In contrast to traditional CNNs, which are constrained by limited receptive fields and focus predominantly on local features, Transformers can analyze spatial patterns across the entire brain. This global perspective allows for the more effective identification of diffuse boundaries and subtle contrasts between lesions and surrounding healthy tissue—challenges that CNNs typically struggle to overcome. By integrating both global contextual information and local details, our approach significantly improves the accuracy and consistency of stroke lesion segmentation. Therefore, the ability to capture long-range dependencies is crucial in enhancing the delineation of stroke lesions from brain imaging, as it facilitates a more comprehensive understanding of the spatial relationships within the data.

The core components of the Transformer model encompass a position encoding module, a multi-head attention mechanism, an Add & Norm strategy, and a feedforward network (FFN).

MHSA: Within a simplistic Transformer model, the self-attention mechanism undertakes the task of transforming an input vector into three distinct vectors; the key (K), value (V), and query (Q) vectors. This transformation is facilitated by three unique weight matrices, denoted as $W_{Q}$, $W_{K}$, and $W_{V}$, corresponding to the conversion of the original vector into its respective *Q*, *K*, and *V* representations.

Multi-head self-attention not only involves the initialization of a set of matrices for *Q*, *K*, and *V*, but it also initializes multiple groups, as illustrated in Figure 3. MHSA, a vital component of the Transformer model, operates uniquely with modular neurons. Through multiple attention heads, the Transformer can effectively recall memory contents during computation, facilitating information transfer to various parts within the context. The pivotal component of the Transformer, known as the MHSA mechanism H, merges distinct self-attention outputs labeled as $H_{i}$, forming its core essence. These outputs are defined by the following concatenation operation:

(1) $$H=Concatenate(H_{1},H_{2},\ldots ,H_{n})$$

where *n* represents the head’s number. The self-attention mechanism enables the gathering of pertinent details among tokens within the sequence. The computation of $H_{i}$ involves the application of the softmax function on the dot product between $Q_{i}$ et $K_{i}^{T}$, followed by element-wise multiplication with $V_{i}$. This process results in a single-head self-attention output. Additionally, the attention matrices $Q_{i}K_{i}^{T}$ are normalized into probability distributions through the softmax function. Intermediate representations $Qi=W_{qi}\tilde{X}$, $Ki=W_{ki}\tilde{X}$, and $Vi=W_{vi}\tilde{X}$ are obtained through linear transformations of input tokens $\tilde{X}$. Here, $W_{qi}$, $W_{ki}$, and $W_{vi}$ represent learned weight matrices for *Q*, *K*, and *V*, respectively. By employing diverse weight matrices, diverse single-head self-attention outcomes can potentially be assembled, allowing for flexible information processing.

Add & Norm strategy: The Add & Norm strategy in Transformers mitigates practical information loss and gradient vanishing issues by employing residual connections and layer normalization. Different performance configurations, such as post-norm, pre-norm, and res-post-norm residual blocks, offer solutions, while the Feed-Forward Network module further enhances the Transformer’s capability by transforming averaged attention values for subsequent layers.

Feed-Forward Network (FFN): The FFN serves as a fundamental component within the Transformer architecture, essential for the efficient processing of input sequences. Following the multi-head self-attention layers, the FFN functions as a position-wise fully connected network. Its design ensures that each position within the input sequence undergoes individual processing while maintaining consistent treatment across positions. This consistency is pivotal for preserving the positional integrity of the input data throughout the transformation process. The FFN comprises dual linear transformation layers complemented by a ReLU activation function applied after the first layer. The absence of the activation function after the second layer simplifies the structure while still enabling effective feature transformation. Mathematically, the FFN’s operations are represented as

(2) $$FFN\left(X\right)=max\left(0,XW_{1}+b_{1}\right)W_{2}+b_{2}$$

where $W_{1}$ and $W_{2}$ are the weight matrices and $b_{1}$ and $b_{2}$ are the biases for the linear layers.

Overall, the FFN plays a crucial role in transforming the mean attention scores into a more practical format, facilitating smoother transitions between layers and enhancing the architecture’s capacity to extract meaningful details from input sequences.

Traditional architectures like U-Net and ResUNet [27] rely on the direct concatenation of encoder and decoder feature maps. Inspired by the effectiveness of attention mechanisms in NLP and computer vision, we propose incorporating an attention block specifically within the decoder portion of our network. This strategic integration enables the model to devote heightened focus to critical areas within the decoder feature maps, leading to more precise segmentation outcomes.

### 3.2. Training Techniques

In the training phase, the initialization of weights plays a crucial role in steering the model toward convergence. We adopt the he-normal initialization method, wherein weights are drawn from a normal distribution truncated and centered around zero, with a standard deviation σ computed as

(3) $$\sigma =\sqrt{\frac{2}{m}},$$

where *m* represents the number of input modules within the weight tensor. This initialization approach aims to establish a balanced starting point for weight updates, promoting smoother convergence and improved model performance.

Additionally, the Adam optimizer is utilized for weight updates, employing a batch size of 8 to enhance computational efficiency. Adam is widely recognized as a highly efficient optimizer in the realm of deep learning methodologies, offering sophisticated mechanisms for dynamically adjusting learning parameters and drawing inspiration from the features of the training dataset.

In the realm of medical image segmentation using deep learning, choosing the optimal loss function is crucial for effective model training.

Binary Cross-Entropy (BCE): A popular choice, BCE penalizes the model for misclassifying pixels (lesion vs. background). However, it can be disadvantaged by imbalanced datasets, where background pixels significantly outnumber lesion pixels. This can lead the model to prioritize learning the background class, neglecting accurate lesion segmentation.
  (4) $$\mathrm{BCE}=-\sum_{i=1}^{M}y\log\hat{y}$$

In this context, $y_{i}$ represents the actual label, ${\hat{y}}_{i}$ signifies the estimated probability, and *M* denotes the number of samples.

Dice Loss: This loss function directly addresses the limitations of BCE. It focuses on the convergence of the anticipated lesion mask and the actual ground truth mask. By rewarding a high degree of overlap, Dice Loss proves more robust to class imbalance, making it a preferred choice for lesion segmentation tasks.
  (5) $$\mathrm{Dice}\;\mathrm{Loss}=-(y-\frac{2|y\cap \hat{y}|}{\left|y\right|\cup |\hat{y}|})$$

Sigmoid BCE: This is a combination of the sigmoid activation function and the BCE loss. The sigmoid function transforms the output of the architecture into probabilities within the range of 0 to 1, representing the likelihood of a pixel being part of the background or the lesion class. While sigmoid BCE can be helpful for interpreting model predictions, it is the BCE component that truly drives the learning process.
  (6) $$\mathrm{Sigmoid}\;\mathrm{Cross}\;\mathrm{Entropy}=-\sum_{i=1}^{M}y_{i}\log\left(\sigma \left({\hat{y}}_{i}\right)\right)$$
  where $\sigma ({\hat{y}}_{i})$ denotes the application of the sigmoid function to the predicted ${\hat{y}}_{i}$, which compresses its input values to the range [0, 1].

Binary Cross-Entropy-Dice (BCE-Dice): The BCE-Dice loss function is a composite metric commonly used in semantic segmentation tasks, particularly in the analysis of medical images. It combines the advantages of the BCE loss and the Dice coefficient, aiming to address the limitations of each individual metric. The BCE component evaluates the pixel-wise binary classification accuracy by comparing the actual ground truth mask to the anticipated lesion mask, penalizing discrepancies between them. On the other hand, the Dice coefficient evaluates the degree of overlap between the predicted masks and the ground truth masks, offering a metric for segmentation accuracy that remains unaffected by class imbalance. By combining these two metrics, the BCE-Dice loss function offers a comprehensive assessment of segmentation performance, effectively capturing both pixel-wise accuracy and spatial overlap. This makes it particularly well suited for tasks where the accurate delineation of regions of interest is critical, such as in medical image segmentation.
  (7) $$\mathrm{BCE}\;\mathrm{Dice}\;\mathrm{Loss}=-\sum_{i=1}^{M}y_{i}\log{\hat{y}}_{i}+\left(y-\frac{2|y\cap \hat{y}|}{\left|y\right|\cup \hat{y}|}\right)$$

In the next section, we will use these loss functions to measure the error, discerning the best loss function for our architecture.

## 4. Data

### 4.1. Data Description

The ISLES datasets are publicly available collections used in stroke segmentation challenges held as part of the International Conference on Medical Image Computing and Computer-Assisted Intervention (MICCAI). These datasets offer an extensive array of MRI sequences, specifically curated to represent various cases of acute and sub-acute stroke.

We selected the ISLES SISS 2015 and ISLES 2022 datasets to explore stroke lesion segmentation over different timeframes. ISLES 2022 builds on the ISLES 2015 dataset by addressing previous limitations through updated challenge standards and the use of a standardized platform, enabling fair comparisons of software solutions. Additionally, ISLES 2022 includes more than six times the number of patients, significantly expanding its scope.

Both datasets present a high degree of lesion variability, including differences in lesion size, location, and the presence of single or multi-focal lesions. This variability is essential for robust evaluations, as it allows segmentation methods to be tested across a broad spectrum of realistic clinical scenarios. The larger patient cohort and standardized evaluation in ISLES 2022 provide a more reliable and comprehensive benchmark, ultimately enhancing the relevance and generalizability of research outcomes.

**Figure 4:**
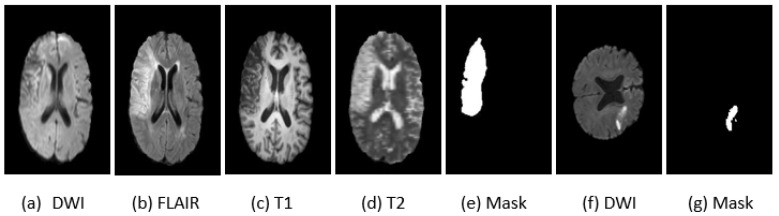
Examples of stroke images and corresponding masks from the SISS 2015 (**a**–**e**) and ISLES 2022 (**f**,**g**) datasets Across various MRI modalities.

The ISLES 2015 challenge dataset, focusing on the sub-acute ischemic stroke image segmentation (SISS) task. This dataset encompasses 64 patient cases, with 28 designated for training and 36 for testing. Each case incorporates a quartet of co-aligned MRI techniques: Fluid-Attenuated Inversion Recovery (Flair), DWI, T1-weighted (longitudinal relaxation time), and T2-weighted (transverse relaxation time) modalities. The MRI images have a resolution of 230 × 230 × 153 voxels, with a voxel spacing of 1 mm in each dimension. Ground truth annotations are provided at the pixel level, enabling a comprehensive evaluation. Notably, the dataset features lesions that are typically small and diffuse, posing a significant challenge for segmentation algorithms. Figure 4 illustrates sample images from these datasets.

ISLES 2022 provides a comprehensive, multicenter MRI dataset specifically designed to aid in segmenting lesions from acute to subacute strokes. This dataset, containing 400 MRI cases collected from multiple vendors, showcases an extensive diversity in stroke lesion sizes, quantities, and locations. To support model training and validation, the dataset is divided into a publicly accessible training set of 250 cases and a testing set of 150 cases reserved exclusively for final validation.

The ISLES 2022 dataset includes crucial MRI sequences: FLAIR, DWI, and the Apparent Diffusion Coefficient (ADC) map, providing a robust foundation for accurate lesion analysis. All images are provided in NIfTI format, ensuring consistency and compatibility across different research tools, with corresponding segmentation masks available for precise ground truth labeling. Examples of these dataset samples are displayed in Figure 4.

### 4.2. Data Preprocessing

To ensure high-quality training data, we implemented a comprehensive preprocessing pipeline. First, images were extracted from the specified modalities, with the SISS 2015 dataset comprising 3D brain scans from 64 subjects. Each scan contained 153 slices, resulting in a total of 9792 2D brain images across all subjects. Recognizing that some slices contain limited information, particularly near the edges of each scan, we identified and removed these blank slices from both ends for each patient. This step refined the dataset to approximately 3900 images per modality, retaining only the relevant data for model training.

The original 230 × 230-pixel resolution of these images posed challenges during the upsampling and downsampling operations. To streamline processing, we resized the images to 192 × 192 pixels, optimizing compatibility with our model’s filter sizes and sampling rates. Next, the image data were normalized to a range of 0 to 1 to support improved model convergence.

This preprocessing pipeline was also applied to the ISLES 2022 dataset, specifically utilizing the DWI modality due to its consistent voxel spacing compared to other modalities like FLAIR. We selected a representative set of 150 preprocessed 2D images from this dataset to perform additional testing, evaluating our model’s generalizability and performance across varied data sources.

**Figure 5:**
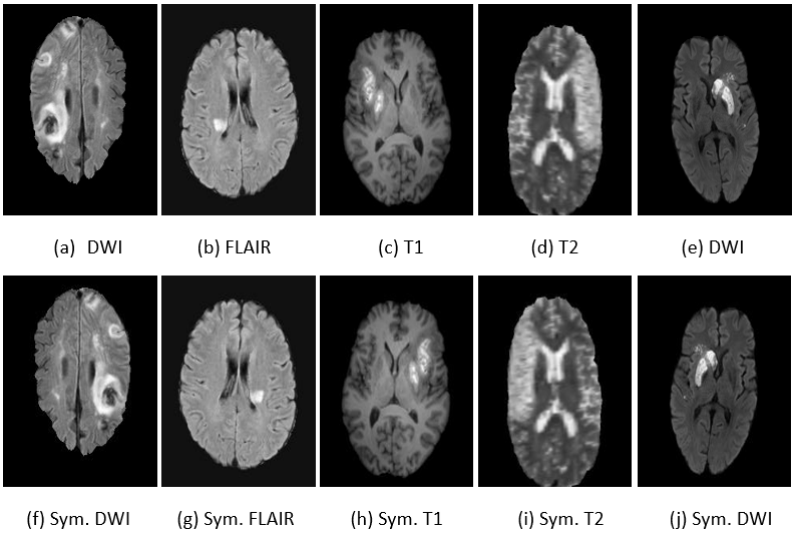
Symmetrically augmented modalities in the ISLES SISS 2015 and ISLES 2022 dataset.

Given the relatively modest size of the preprocessed SISS 2015 dataset, we expanded it through data augmentation, employing methods such as horizontal flipping, zooming, shearing, and rotation (with rotation angles randomly selected between 0 and 90 degrees). This augmentation process increased the dataset to approximately 11,700 images, addressing limitations posed by a smaller dataset size—an often-encountered challenge in medical image analysis. Figure 5 presents examples of symmetrically augmented images from both datasets. Subfigures (a), (b), (c), (d), (f), (g), (h), and (i) correspond to various modalities from the SISS 2015 dataset, while subfigures (e) and (j) depict the DWI modality and its symmetrical counterpart from the ISLES 2022 dataset.

Following preprocessing and augmentation, the data were randomized and split, with 80% allocated for training and 20% reserved for validation. This comprehensive approach to data preparation ensured robustness and improved adaptability for neural network training, making it especially suited for medical imaging tasks.

## 5. Evaluation Metrics

In the domain of segmentation, judiciously chosen evaluation metrics are essential for gauging the efficacy of various algorithms. As the quality of segmentation increases, the importance of each metric becomes more prominent. These metrics are meticulously crafted to facilitate objective comparisons between different segmentation outputs, ensuring a consistent evaluation process. Notably, these metrics produce decimal values between 0.0 to 1.0, where a value closer to 1.0 signifies near-perfect and complete overlap between the anticipated segmentation and the actual ground truth. We evaluated our model’s performance using two frequently utilized quality metrics, with each defined as follows.

### 5.1. Dice Coefficient

A fundamental metric in evaluating segmentation models, the Dice similarity coefficient provides crucial insights into the alignment among anticipated segmentation and real ground truth data.

(8) $$Dice=\frac{2TP}{2TP+FP+FN}$$

### 5.2. Accuracy

This crucial assessment metric plays a pivotal part in thoroughly evaluating the performance of a classification model. It gauges accuracy through the comparison between accurately predicted instances and the total instances under evaluation, thus providing deep insights into the model’s efficacy.

(9) $$Accuracy=\frac{TP+TN}{TP+TN+FP+FN}$$

where the four fundamental terms FP (False Positive), FN (False Negative), TN (True Negative), and TP (True Positive) represent distinct scenarios. A FN occurs when lesion regions are mistakenly classified as non-lesion regions. Conversely, a FP indicates non-lesion areas that are incorrectly identified as lesions. A TP represents accurately classified lesion regions, while a TN signifies correctly identified non-lesion areas.

## 6. Experimental Results and Discussion

To implement our model, we used the Keras framework, a deep learning wrapper library with a TensorFlow backend [28] within a Conda environment. The training of the proposed architecture was performed on Google Colab Pro, utilizing a system with 51 GB of RAM. Our model requires approximately one hour of training on an NVIDIA Tesla T4 GPU. Our model has a total of 13,662,913 parameters (approximately 52.12 MB), with 13,649,473 trainable parameters (52.07 MB) and 13,440 non-trainable parameters (52.50 KB). This parameter count reflects the increased complexity introduced by the Transformer blocks and dilated convolutions.

As highlighted earlier, our Transformer Dil-DenseUNet network incorporates essential components for enhanced performance. This includes integrating a dilated block before each Dense Block to form the Dilated Dense Block (DDB) within the DenseUNet architecture. Additionally, the decoder part utilizes multi-head self-attention through attention blocks. The proposed network was separately experimented with the Flair, DWI, T1, and T2 MRI modalities.

The results obtained for four standard loss functions, as detailed in Table 1, span both the training and testing phases, indicating robust learning and adaptation. Notably, among the various imaging modalities, the DWI modality consistently demonstrates efficiency and robustness for stroke image segmentation, irrespective of the loss function employed to evaluate the architecture. Furthermore, the analysis of the same table suggests that the BCE Dice Loss function outperforms the other four loss functions.

Based on the insights gleaned from Table 1, we have decided to proceed with the DWI dataset due to its proven track record in delivering high-quality images. Additionally, the BCE Dice Loss function was chosen as a pivotal metric for evaluating errors and guiding model optimization within this architecture. Minimizing this loss function during training facilitates parameter refinement and enhances segmentation accuracy, thus serving as the primary objective of the training process.

**Figure 6:**
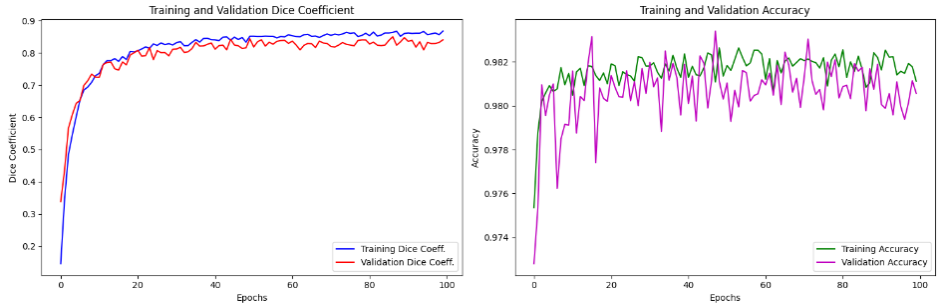
Visual representations of Dice coefficient and Accuracy plots for the DWI modality, showcasing both training and validation sets, arranged from left to right.

To ensure efficient evaluation and model effectiveness, the careful selection of hyperparameter values was essential and is summarized in Table 2. Initial values for key parameters, including the learning rate, batch size, and dropout rate, were informed by standard practices in deep learning and adapted to meet the specific requirements of medical image segmentation. The learning rate was set at $10^{-4}$, a commonly recommended value that supports stable convergence, particularly in complex architectures. We used the Adam optimizer for its adaptive learning rate, well-suited to the high-dimensional nature of MRI data. Given computational constraints, we explored dropout rates from 0.1 to 0.3 and batch sizes of 4, 8, and 16. Ultimately, a dropout rate of 0.2 and a batch size of 8 achieved the optimal balance of training stability and memory efficiency within the limitations of Google Colab Pro. These choices were guided by preliminary experiments and validated by observing metrics such as the Dice coefficient and accuracy, which helped ensure optimal generalization and minimized overfitting. Figure 6 presents the resulting Dice coefficients and accuracy metrics during training and validation for the Transformer Dil-DenseUNet model.

**Figure 7:**
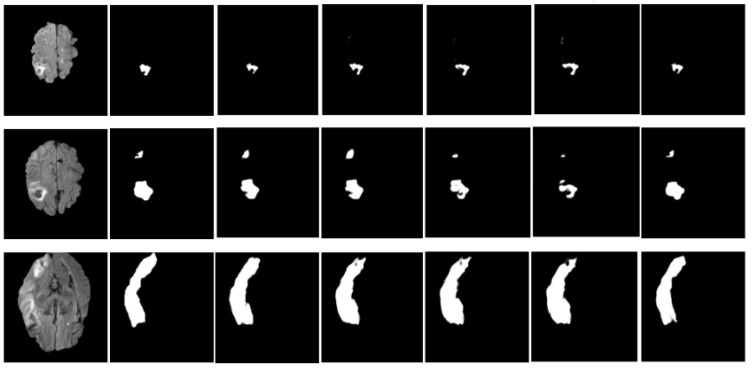
Comparison of qualitative results on the SISS 2015 dataset. From left to right: (1) input image, (2) ground truth, (3) Transformer DenseUNet, (4) Dil-DenseUNet, (5) DenseUNet, (6) Transformer UNet, and (7) Transformer Dil-DenseUNet. Experimental results indicate that Transformer Dil-DenseUNet produces more accurate segmentation masks than the other models.

**Figure 8:**
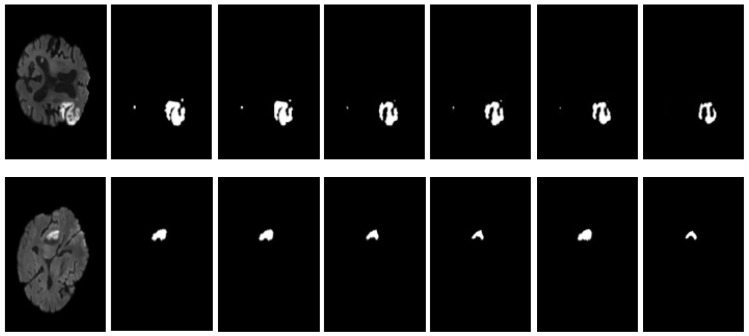
Comparison of qualitative results on the ISLES 2022 dataset. From left to right: (1) input image, (2) ground truth, (3) Transformer DenseUNet, (4) Dil-DenseUNet, (5) DenseUNet, (6) Transformer UNet, and (7) Transformer Dil-DenseUNet. Experimental results indicate that Transformer Dil-DenseUNet produces more accurate segmentation masks than the other models.

The remarkable effectiveness of our proposed model is clearly demonstrated by the qualitative results on the SISS 2015 and ISLES 2022 datasets, as shown in Figure 7 and Figure 8, respectively. Each row presents an individual patient case, showcasing brain slices in the axial view, with columns from left to right: (1) input image, (2) ground truth, (3) Transformer DenseUNet, (4) Dil-DenseUNet, (5) DenseUNet, (6) Transformer UNet, and (7) Transformer Dil-DenseUNet.

The first row displays a small lesion at the brain’s edge, the second row shows multifocal lesions with indistinct boundaries, and the third row highlights a large, complex lesion. Our model excels in capturing intricate details and produces more accurate segmentation masks than other models, consistently achieving high performance. These results underscore the robustness and precision of our architecture in segmenting diverse lesion characteristics.

In the ablation study presented in Table 3, we evaluate five model configurations: Transformer DenseUNet, Dil-DenseUNet, DenseUNet, Transformer UNet, and our proposed Transformer Dil-DenseUNet. This study aims to elucidate the unique contributions of each architectural component to the overall performance of our model. The results reveal that the Transformer Dil-DenseUNet achieves superior performance, with a Dice coefficient of 0.86 during training and 0.80 during testing. This significant improvement demonstrates the model’s enhanced ability to accurately delineate stroke lesion boundaries, which is critical for effective segmentation in medical imaging. Furthermore, it reaches 98% accuracy in training and 94% in testing, surpassing all other configurations and underscoring its strong generalization capabilities on both familiar and unseen data.

Each architectural component contributes uniquely to the model’s robustness. The inclusion of DenseNet promotes feature reuse, improving precision in capturing detailed stroke lesion characteristics. Its dense connections mitigate the vanishing gradient problem, facilitating stable training and better extraction of fine-grained details in brain MRIs. The ablation results clearly indicate that configurations lacking DenseNet exhibit lower performance, highlighting its importance in refining segmentation quality.

Additionally, the integration of dilated convolutions extends the receptive field without increasing computational complexity, allowing the model to gather more contextual information. This feature is particularly advantageous for segmenting irregular and diffuse stroke lesions. Configurations without dilated convolutions show lower accuracy and Dice scores, reinforcing their necessity in capturing broader context while maintaining local details.

The multi-head self-attention mechanism of the Transformer is crucial for capturing long-range dependencies across the entire image. This capability is essential for accurately delineating lesion boundaries, especially in complex cases with low contrast or diffuse edges. Models lacking Transformer components demonstrate lower segmentation accuracy, emphasizing the importance of this mechanism in improving overall performance.

In contrast, configurations such as DenseUNet and Transformer UNet, which do not incorporate either the Transformer or dilated convolutions, yield inferior performance. For instance, Transformer UNet achieves a testing Dice of 0.70 and an accuracy of 0.88, highlighting the necessity of integrating DenseNet with Transformer and dilated convolutional enhancements for superior segmentation quality.

In conclusion, this ablation study validates the contributions of each component within our proposed Transformer Dil-DenseUNet. The findings demonstrate how the integration of these elements culminates in robust segmentation performance, significantly advancing methods for accurate stroke lesion delineation in medical imaging.

To assess the performance of our proposed model, we calculated the Dice coefficient and compared it with state-of-the-art methods on the SISS dataset. The comparative results are presented in Table 4. A closer look at these findings reveals that our model outperforms all other methods in terms of the Dice score. Specifically, our model achieved an average Dice score of 0.80 ± 0.30 on the SISS 2015 testing dataset, which is a significant improvement over the best result from previous studies.

For instance, Liu et al. [15] reported a Dice score of 0.76 ± 0.31, while Zhang et al. [29] obtained a lower score of 0.63. Additionally, when applied to the ISLES 2022 dataset, our model further demonstrated its robustness, achieving a Dice score of 0.81 ± 0.33. These results highlight the superior performance of our architecture in the task of stroke lesion segmentation, showcasing its ability to handle various challenges within the dataset and outpacing existing methods in accuracy and consistency.

Overall, the obtained results demonstrate promising performance, indicating the potential of our model to accurately delineate stroke lesions across different imaging modalities. Notably, the implementation of our model requires only 2 h, which significantly decreases computational demands and enhances its scalability for deployment in real-time clinical settings.

One of the primary advantages of our proposed model is its capacity to capture both local and global contextual information effectively. By integrating dense connectivity patterns from DenseNet, dilated convolutions, and Transformer mechanisms, our model achieves robust segmentation results. The dense connectivity allows for feature reuse, while dilated convolutions enlarge the receptive field without significantly augmenting computational complexity. The Transformer mechanism enables the model to capture long-range dependencies, enhancing its ability to discern significant features within the input data.

Furthermore, our model shows versatility in handling different imaging modalities, with particularly noteworthy performance in the DWI modality. This underscores the effectiveness of our approach and its potential for real-world clinical applications.

Despite the promising results, our study has a variety of limitations that warrant discussion. The performance of our model may be influenced by factors such as dataset size, image quality, and variability in stroke lesion characteristics. While we have attempted to address these challenges through data preprocessing and augmentation techniques, further exploration is necessary to assess the extent to which our model demonstrates generalization capabilities across various imaging modalities and conditions.

## 7. Conclusions

This study presented the Transformer Dil-DenseUNet, a novel architecture designed for stroke lesion segmentation in MRI images. By integrating DenseNet, dilated convolutions, and Transformer blocks, our model effectively captures both detailed local features and essential long-range dependencies. The DenseNet component ensures feature reuse and fine-grained detail extraction, while dilated convolutions enhance the receptive field, enabling the capture of broader contextual information. The Transformer blocks overcome the limitations of conventional CNNs by modeling global relationships across the image, thus improving segmentation precision, especially for complex lesion boundaries.

Our model was rigorously evaluated on the ISLES 2015 and ISLES 2022 datasets, where it achieved superior Dice coefficients and segmentation accuracy compared to existing models. The results highlight the architecture’s robustness across different imaging modalities and its ability to adapt to the diverse characteristics of stroke lesions. Additionally, ablation studies demonstrated the distinct contributions of each component in improving the overall segmentation performance.

Future work could focus on enhancing the architecture with advanced post-processing techniques and multimodal data integration (e.g., CT or PET imaging). Additionally, extending the model for 3D image data and adapting it for other neurological conditions could broaden its impact, advancing automated lesion segmentation in medical imaging.

## Figures and Tables

**Table 1:** Assessing the efficacy of the suggested model across different loss functions by measuring the Dice coefficient for each modality within the SISS Dataset.

Modality	SCE		Dice Loss		BCE		BCE Dice Loss	
	Training	Testing	Training	Testing	Training	Testing	Training	Testing
DWI	0.78	0.66	0.85	0.74	0.84	0.75	0.86	0.80
Flair	0.80	0.68	0.80	0.69	0.81	0.72	0.83	0.75
T1	0.76	0.66	0.77	0.68	0.78	0.67	0.80	0.74
T2	0.71	0.63	0.76	0.69	0.75	0.64	0.80	0.73

**Table 2:** Hyper-parameters of the Transformer Dil-DenseUNet.

Hyper-Parameter	Value
Kernel	(3 × 3)
Batch-size	8
Activation function	ReLU
Dropout	0.2
Learning Rate	$10^{-4}$
Optimizer	Adam
Epochs	100

**Table 3:** Ablation study of the proposed model evaluating accuracy and the Dice coefficient across five configurations: Transformer DenseUNet, Dil-DenseUNet, DenseUNet, Transformer UNet, and Transformer Dil-DenseUNet.

Configuration	Dice		Accuracy	
	Training	Testing	Training	Testing
Transformer DenseUNet	0.84	0.76	0.97	0.90
Dil-DenseUNet	0.85	0.73	0.95	0.84
DenseUNet	0.83	0.72	0.93	0.90
Transformer UNet	0.82	0.70	0.92	0.88
Transformer Dil-DenseUNet	0.86	0.80	0.98	0.94

**Table 4:** Analyzing Dice scores across different techniques on the SISS dataset, with averages computed over the entire patient cohort.

Method	Testing
Zhang et al. [29]	0.63
Kumar et al. [16]	0.63±0.25
Dogru et al. [21]	0.74±0.01
Alshehri et al. [30]	0.76±0.02
Liu et al. [15]	0.76±0.31
Our Model with SISS 2015	0.80 ± 0.30
Our Model with ISLES 2022	0.81 ± 0.33

## Data Availability

The data included in this manuscript cannot be shared publicly, due to the need to protect the privacy of the included subjects. Data may be shared upon reasonable request to the corresponding author.
